# Open reduction and internal fixation of displaced head-split type humeral fractures and role of the rotator-interval approach

**DOI:** 10.1177/17585732211065449

**Published:** 2021-12-15

**Authors:** E Fleischhacker, G Siebenbürger, J Gleich, T Helfen, W Böcker, B Ockert

**Affiliations:** Department of Orthopaedics and Trauma Surgery, Musculoskeletal University Center Munich (MUM), University Hospital, 9183 LMU Munich, Munich, Germany

**Keywords:** humerus fracture, head-split, osteosynthesis, avascular necrosis, constant-score

## Abstract

**Background:**

Open reduction and internal fixation (ORIF) of humeral head split fractures is challenging because of high instability and limited visibility. The aim of this retrospective study was to investigate the extend of the approach through the rotator interval (RI) on the reduction quality and functional outcome.

**Methods:**

37 patients (mean age: 59  ±  16 years,16 female) treated by ORIF through a standard deltopectoral (DP) approach were evaluated. The follow-up period was at least two years. In 17 cases, the approach was extended through the RI. Evaluation was based on radiographs, Constant scores (CS) and DASH scores.

**Results:**

In group DP, “anatomic” reduction was achieved in 9 cases (45%), “acceptable” in 5 cases (25%), and “malreduced” in 6 cases (30%). In group RI, “anatomic” reduction was seen in 12 cases (71%), “acceptable” in 5 cases (29%), and “malreduced” in none (p  =  0.04). In the DP group, the CS was 60.2  ±  16.2 and the %CS was 63.9  ±  22.3, while in the RI group, the CS was 74.5  ±  17.4 and the %CS was 79.1  ±  24.1 (p  =  0.07, p  =  0.08). DASH score was 22.8  ±  19.5 in DP compared to RI: 25.2  ±  20.6 (p  =  0.53).

**Conclusions:**

The RI approach improves visualization as it enhances quality of fracture reduction, however functional outcomes may not differ significantly.

**Type of study and level of proof:**

Retrospective, level III

## Background

Proximal humeral fractures account for 5-10% of all fractures.^1,2^ Although the incidence is high, especially in the elderly population, it is controversial what is the best treatment.^3,4^ While non-displaced fractures can be treated conservatively, for displaced fractures, particularly in younger patients, operative treatment remains an option.^4–7^ Open reduction and internal fixation (ORIF) is the most frequently performed procedure in the surgical treatment of proximal humeral fractures,^3,5,6,8^ however, in complex fracture patterns, such as head-split fractures, some authors suggest primary anatomic or reverse arthroplasty, due to considerably high complication rates of up to 50%.^4,5,8^

Split type fractures of the humeral head are believed to be challenging fractures in terms of accurate fracture reduction, fracture healing and the risk of avascular necrosis.^4,9^ The head-split fragment is often shallow, offering limited opportunity for internal fixation.^10^ As a consequence, it may be difficult to achieve anatomic reduction, particularly due to limited visibility of the articular surface.^9^ Nevertheless, anatomic reduction is believed to be an important factor for the fracture to heal and in order to prevent avascular necrosis of the humeral head.^11^ One option to improve visibility and potentially increase the quality of fracture reduction may be by limited extension through the rotator interval (RI), between the supraspinatus and subscapularis tendon. However, its impact on the quality of reduction in head-split type fractures is unreported.

The purpose of this study was to evaluate the quality of fracture reduction in humeral head-split type fractures treated by ORIF, utilized by additional visualization through a RI approach, in comparison to fractures that were reduced through a standard DP approach only. We hypothesized, that additional exposure may help to improve the quality of fracture reduction. In addition, we aimed to compare surgical approaches with regards to fracture union, fixation failure and avascular necrosis, as well as functional outcomes.

## Patients and methods

### Study cohort

**Figure 1.**
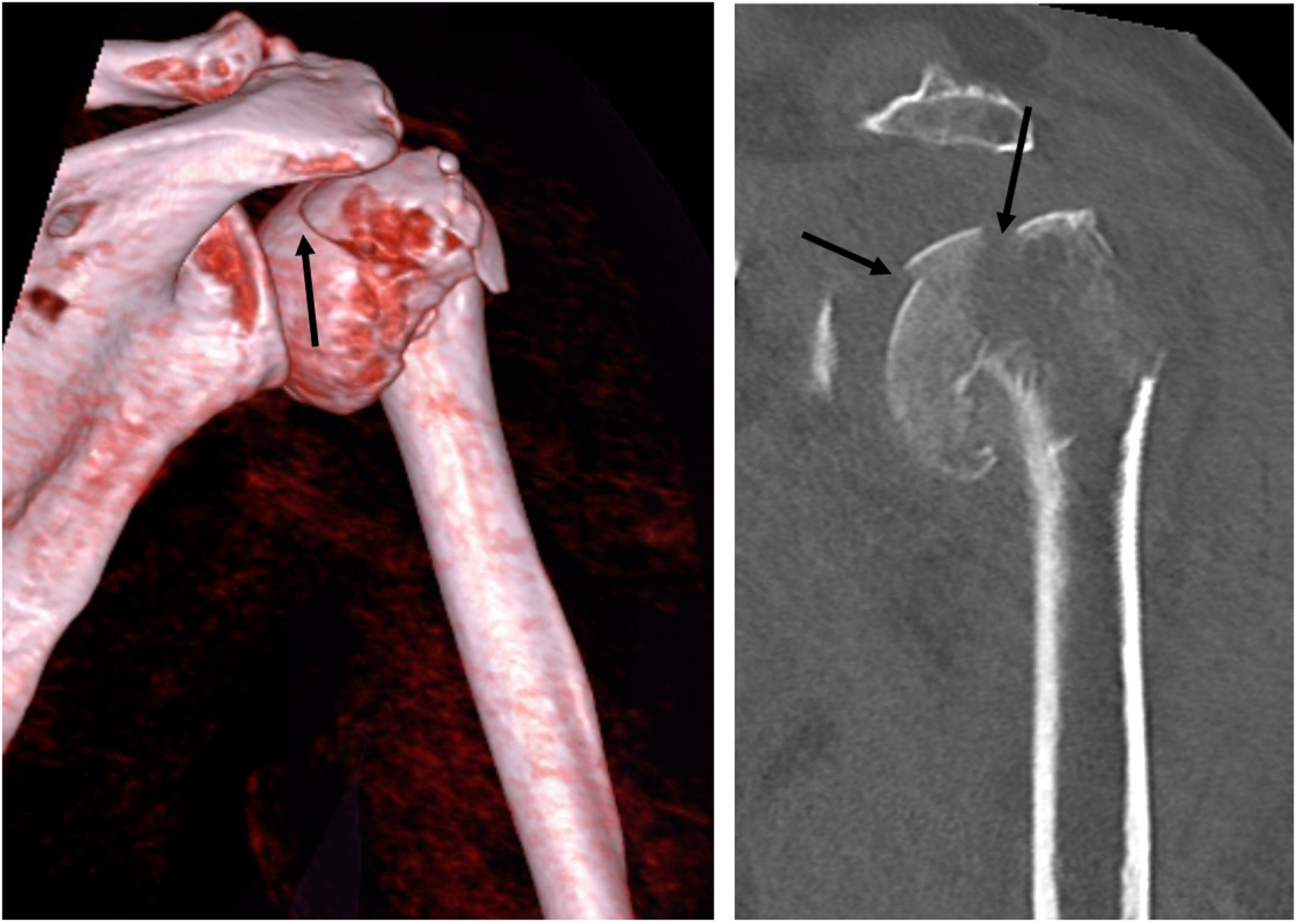
Ct images of patients with headsplit fractures included in the study: dislocated (>5 mm) fractures (arrows) located >5 mm within the cartilaginous articular surface. Left, 3D reconstruction of a CT scan; right, a coronal slice.

This retrospective study was approved by the local ethical review committee (156-12), planned and conducted according to the declaration of Helsinki. Patient data and records of all head-split type proximal humerus fractures, that were treated by ORIF between November 2006 and December 2017, were retrieved. Fractures were assessed by standard anteroposterior and lateral-view x-rays as well as 2D and 3D computer tomography (CT) scans in all cases. We defined a true head-split type proximal humeral fracture as a fracture within the humeral head cartilage according to the early description by Charles Neer.^10^ Therefore, fractures were excluded, that were not characterized by a displaced (>5 mm) true split of the humeral head >5 mm within the cartilage (Figure 1). Furthermore, all fracture dislocations were excluded as these injuries represent a different entity, as were pathologic fractures due to neoplasms. All head-split fractures were treated by locked plating within five days from trauma by four different surgeons. The RI approach was performed upon the surgeons` personal preference for visualization of the fracture. Patients were followed until clinical and radiographic union had occurred, or for a minimum of two years. 47 patients were included according to the above criteria, two patients died, and eight were lost to follow-up. Data of 37 patients (16 female, mean age: 59  ±  16 years (range 26 to 72) with a true head-split type proximal humeral fracture were evaluated. All head-split fractures were classified according to the criteria described by Scheibel et al.^12^ The mean fracture displacement from CT-scans was 8.4  ±  0.9 mm. A summary of the baseline characteristics is given in Table 1.

**Table 1.** Summary of the patients` baseline characteristics.

	**DP (deltopectoral) n = 20**	**RI (rotator interval) n = 17**	**p value**
**AGE (YR)**	59.1 ± 13.4	58.7 ± 15.1	0.36
**SEX (% FEMALE)**	9 (56%)	7 (44%)	0.41
**SMOKING (YES)**	4 (20%)	5 (29%)	0.74
**MEAN ASA**	2.3 ± 0.7	2.2 ± 0.5	0.87
**FRACTURE TYPE (SCHEIBEL^12^)**			0.60
** I**	7 (35%)	7 (41%)	
** II**	8 (40%)	7 (41%)	
** III**	3 (15%)	3 (18%)	
** IV**	2 (10%)	0	
**DISPLACEMENT IN MM**	7.6 ± 0.7	9.3 ± 1.2	0.34
**QUALITY OF REDUCTION**			0.04
** “ANATOMIC”**	9 (45%)	12 (71%)	
** “ACCEPTABLE”**	5 (25%)	5 (29%)	
** “MALREDUCED”**	6 (30%)	0 (0%)	
**CONSTANT SCORE**	60.2 ± 16.2	74.5 ± 17.4	0.07
**CONSTANT SCORE %**	63.9 ± 22.3	79.1 ± 24.1	0.08
**DASH SCORE**	25.2 ± 20.6	22.8 ± 19.5	0.53
**SEC. DISPL. / NON-UNION**	1 (5%)	1 (6%)	1.00
**AVN**	4 (20%)	3 (18%)	0.71

ASA: American Society of Anaesthesiologists; AVN: avascular necrosis; MM: millimeters; Sec. Displ.: secondary displacement; YR: Years.

### Surgical procedure

The surgery was performed with the patient in beach chair position on a fracture table. Every patient received prophylactic intravenous antibiotics (Cefuroxime^®^) 1,5g single-shot and general anesthesia in combination with an interscalene block for pain control.

### Deltopectoral only vs. Rotator interval approach

**Figure 2.**
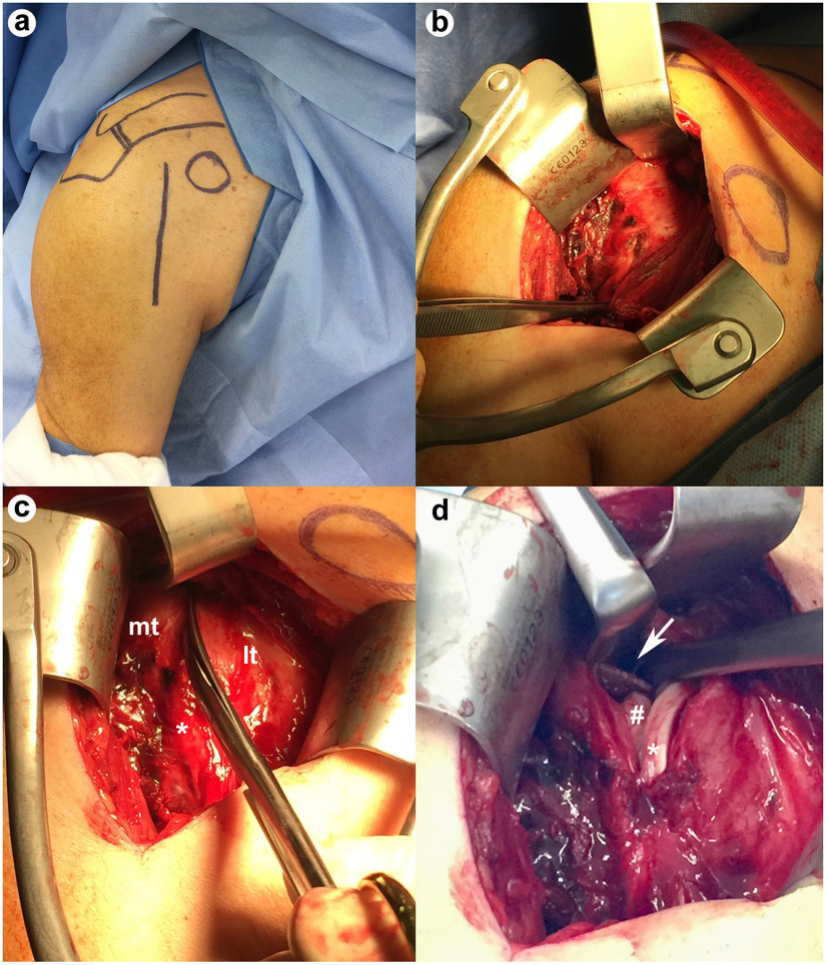
Establishing the rotator interval approach

In 20 cases the fracture was reduced and fixed through a standard deltopectoral approach only (**DP**). In five of these 20 cases a fracture of the major tubercle was followed into a longitudinal split of the supraspinatus tendon. In comparison, in 17 cases, a supplementary rotator interval approach (**RI**) was established between the supraspinatus and subscapularis tendon in order to improve visualization and utilize for fracture reduction (Figure 2). The biceps tendon was then cut at the supraglenoid tubercle and a biceps tenodesis was performed to the pectoralis major tendon-muscle junction.

a) right shoulder with bony landmarks and marked skin incision for a standard deltopectoral approach.

b) Insertion of a self-retaining retractor between the deltoid and pectoralis major muscle showing the humeral head with intact rotator cuff; forceps indicating the tendon of the long head biceps.

c) Opening the rotator interval between the supraspinatus tendon and the subscapularis tendon beginning from the bicipital groove between major (mt) and lesser tubercle (lt) following the tendon of the long head biceps (*) to the supraglenoid tubercle.

d) Inspection of the glenohumeral joint via the rotator interval approach. Blunt retraction of the long head biceps tendon (*) with elevator and the supraspinatus tendon by use of a small Langenbeck’s retractor for direct reduction of the humeral head; large posterior fragment (arrow) and anterior “shield” fragment including lesser tubercle in continuity with bicipital groove (#).^22,23^

### Fracture fixation

In all cases, a proximal humeral interlocking plate system (PHILOS^®^, DePuy Synthes, Johnson & Johnson Medical, Raynham, Massachusetts, USA) was used with solid fixation by angular stable screws as previously reported [5,8]. In six cases autogenic bone grafts and in four cases allogenic bone grafts were used to fill defects within the humeral head. In six cases synthetic bone substitutes were used as a void filler and in another two cases bone cement was used around screw tips to increase fixation strength. Use of augmentation was evenly distributed between groups. The rotator cuff was evaluated for rotator cuff tears and tuberosity sutures (FiberWire No. 5; Arthrex, Naples, Florida (FL), USA) were used when necessary. In twenty-two cases, additional interfragmentary screws were inserted from anterior to posterior in order to increase stability by compression of the major fragment to the remaining humeral head. The post-surgical rehabilitation protocol allowed passive and active-assisted rehabilitation exercises followed by active exercises with full range of motion as reported in prior studies.^5,8^

### Radiographic evaluation

Postoperative radiographs included standard anteroposterior and lateral-view x-rays. Quality of fracture reduction was defined according to Schnetzke et al.^11^ By these criteria, a minor varus head-shaft alignment of <120° to 110° or a minor displacement of the greater tuberosity ( ≤ 5 mm) was considered an “acceptable” result of fracture reduction. A head-shaft alignment of <110° or >150° or a displacement of >5 mm was rated as “malreduction”. According to the quantitative determination of fracture reduction, patients were assigned to 1 of 3 groups: overall “anatomical” fracture reduction (achievement of all 3 parameters of anatomical reduction), “acceptable” fracture reduction (achievement of 1 to 3 parameters of acceptable fracture reduction with no parameter of malreduction), and “malreduced” fracture (1 to 3 parameters of malreduction).^11^ Radiographs were used to determine complications such as secondary displacement, non-union and avascular head necrosis at every follow-up visit.

### Follow-up and outcome measures

Outcome measures included the Constant score (CS) for the injured and contralateral shoulder as well as the Disability of the Arm, Shoulder and Hand (DASH) questionnaire. The Constant score was accomplished as a raw value and in percentage to the uninjured side (%CS).

### Statistical analysis

Continuous variables were described using means and standard deviation. Results were compared depending on the surgical approach (**DP** vs. **RI**) using the Mann-Whitney test for continuous variables and the chi2-Test was performed for comparison of dichotomous variables between groups. The level of significance was set at p < 0.05 for all testing. Statistical analysis was performed using SPSS version 22 (SPSS Inc.).

## Results

28 (76%) of 37 fractures united. Two patients had a nonunion and seven patients developed avascular necrosis (AVN) of the humeral head. In 21 cases (57%) the reduction was “anatomic”, in 10 cases (27%) “acceptable” and in 6 cases (16%) the fracture was rated “malreduced”. All fractures that were reduced “anatomically” and 7 fractures of which were reduced “acceptably” healed (p  =  0.47). All fractures that were “malreduced” and 3 cases that were reduced “acceptably” developed secondary displacement, non-union or avascular necrosis. There were no infections or wound complications.

In patients where the head-split fracture was addressed through the deltopectoral approach only (**DP**), “anatomic” reduction was achieved in 9 cases (45%). In 5 cases (25%) the quality of reduction was “acceptable” and in 6 cases (30%) the fracture was “malreduced”. In comparison, by utilizing the rotator interval approach (**RI**) in 12 cases (71%) an “anatomic” reduction was achieved. In 5 cases (29%) the reduction was “acceptable”, while there was no case of a “malreduced” fracture (p  =  0.04, table 1).

The overall mean Constant-score was 68.4  ±  18.2 and the Constant-score% was 71.1  ±  24.5. In patients with an “anatomic” or “acceptable” reduction, the CS was 72.4  ±  18.2 and the Constant-score% was 76.3  ±  22.4, while in patients with “malreduction” the CS was 61.5  ±  15.1 and the Constant-score% was 64.3  ±  21.4 (p  =  0.69). In patients of group **DP** the CS was 60.2  ±  16.2 and the Constant-score% was 63.9  ±  22.3, while in patients of group **RI** the CS was 74.5  ±  17.4 and the Constant-score% was 79.1  ±  24.1 (p  =  0.07, p  =  0.08). The DASH-score was **DP**: 22.8  ±  19.5 compared to **RI**: 25.2  ±  20.6 (p  =  0.53).

Secondary displacement, non-union and avascular necrosis were evenly distributed between the groups (p  =  0.71, p  =  1, Table 1) and were treated by anatomic hemi-endoprosthesis in two cases and reversed shoulder arthroplasty in five cases, while in two cases hardware removal without humeral head replacement was obtained.

## Discussion

This study describes the outcomes following ORIF of head-split type humeral fractures in a larger cohort of patients. Though it can be difficult to achieve anatomic reduction of head-split fractures, the results indicate that anatomic reduction allows for fracture healing and provides satisfying functional outcomes. To facilitate anatomic fracture reduction, the RI approach may be seen as a supplementary option to increase visualization and ensure accurate fracture reduction in a displaced head-split type humeral fracture.

Fixation with humeral locking plate systems has resulted in high union rates and satisfying clinical results.^5,6,8,9,13,14^ Nevertheless, ORIF of humeral head-split type fractures can be technically challenging due to displacement of the fragments, distracting forces of the rotator cuff and limited visibility of the articular surface.^5,9,15^ As a result of our frustration we began to establish a supplementary RI approach, between the supraspinatus and subscapularis tendon in order to facilitate for anatomic reduction, especially the articular surface of the humeral head.

We believe that the union rate of 74% is comparable to reports in the literature for these problematic fractures, despite the routine use of an additional RI approach.^13,14,16^ Although many have discouraged the use of ORIF with locking plate systems for head-split type fractures because of a fear of humeral head necrosis,^9,17,18^ the results in our series demonstrate, when applied in a biologically friendly manner, reduction can be achieved anatomically without adversely affecting bony healing. It is plausible that the excellent osseous apposition provided by the increased visualization outweigh the risks of the additional soft-tissue trauma. In general, visualization may also be improved when extending deep dissection longitudinally in line with the supraspinatus tendon fibres at the level of the head-split fracture site.^18^ However, injury to the important anterolateral vascular branch of the anterior circumflex artery must be avoided by any means, as it may increase the risk of avascular necrosis of the humeral head. Therefore, we recommend opening the RI along the biceps tendon sheath not lower than the subscapularis insertion to prevent injury to the ascending artery.^19,20^ Biceps tendon management (i.e. tenotomy, tenodesis) appears to be important when considering opening of the RI for fracture reduction. Takedown of the biceps tendon may further improve visualization.^21^ However, from this study one may not conclude on the necessity of biceps tendon management overall.

The high rate of anatomic reduction in this series is noteworthy but not surprising given the fact that the fractures were reduced under direct visualization and then fixed by locking plate systems. Although standardized fluoroscopic visualization in perpendicular planes is recommended,^8^ it is our believe that direct visualization is specifically helpful when aiming for anatomic reduction in head-split-type fractures. This is also shown by the fact that despite greater dislocation of the fracture, none of the fractures in the RI group were classified as “malreduced” postoperatively.

The comparison of the functional results between the groups does not show a statistical significance with regard to the Constant score, however, a tendency towards significance at a p  =  0.07 is indeed apparent. Overall, the results of the Constant score evaluation in the RI group are conclusive with the scores reported in the literature, tending to be even slightly better. The DASH shows higher scores in both groups than in comparable studies.^5,6^

The weaknesses of this study include its retrospective nature. Additionally, information on operative time and blood loss was not available in sufficient detail to allow for comparison between standard deltopectoral approach only and supplementary RI approach. However, when dissection is conducted with respect to anatomical structures, we believe, that extension into the rotator-interval results in little blood loss and can be performed quickly for the benefits of a better visualization in the treatment of head-split type fractures.

## Conclusions

ORIF of head-split type humeral fractures remains challenging. However, the results of this study indicate, when anatomic reduction is achieved, fracture healing and functional outcomes are satisfying. Dissection through the RI may be used to improve visualization of head-split type fractures, as it increases the quality of fracture reduction, without increasing the risk of avascular necrosis. The results of this study also show, that utilizing this approach, functional outcomes are higher, however, not significant.

## List of abbreviations

ORIF: open reduction and internal fixation
CS: Constant score
%CS: Percentage Constant Score to the uninjured side
CT: Computer tomography
DASH: Disability of the Arm, Shoulder and Hand questionnaire
DP: deltopectoral
PHILOS: Proximal humeral interlocking plate system
RI: rotator interval
